# Functional lung imaging using novel and emerging MRI techniques

**DOI:** 10.3389/fmed.2023.1060940

**Published:** 2023-04-25

**Authors:** Chuan T. Foo, David Langton, Bruce R. Thompson, Francis Thien

**Affiliations:** ^1^Department of Respiratory Medicine, Eastern Health, Melbourne, VIC, Australia; ^2^Faculty of Medicine, Nursing and Health Sciences, Monash University, Melbourne, VIC, Australia; ^3^Department of Thoracic Medicine, Peninsula Health, Frankston, VIC, Australia; ^4^Melbourne School of Health Science, Melbourne University, Melbourne, VIC, Australia

**Keywords:** fluorinated gas, hyperpolarized gas, magnetic resonance imaging, oxygen-enhanced, technique, perfusion, phased-resolved functional lung imaging, ventilation heterogeneity

## Abstract

Respiratory diseases are leading causes of death and disability in the world. While early diagnosis is key, this has proven difficult due to the lack of sensitive and non-invasive tools. Computed tomography is regarded as the gold standard for structural lung imaging but lacks functional information and involves significant radiation exposure. Lung magnetic resonance imaging (MRI) has historically been challenging due to its short T2 and low proton density. Hyperpolarised gas MRI is an emerging technique that is able to overcome these difficulties, permitting the functional and microstructural evaluation of the lung. Other novel imaging techniques such as fluorinated gas MRI, oxygen-enhanced MRI, Fourier decomposition MRI and phase-resolved functional lung imaging can also be used to interrogate lung function though they are currently at varying stages of development. This article provides a clinically focused review of these contrast and non-contrast MR imaging techniques and their current applications in lung disease.

## Introduction

Respiratory diseases impose an immense worldwide health burden (1–5). As a global measure, traditional lung function tests such as spirometry, e.g., forced expiratory volume in 1 s (FEV_1_) are insensitive to early stage disease, regional heterogeneity, and subtle changes over time. Despite these limitations, spirometry and FEV_1_ remain the default clinical standard for the diagnosis and assessment of various lung diseases such as asthma and chronic obstructive pulmonary disease (COPD), as well as an intermediate endpoint in longitudinal studies and clinical trials. Traditional high resolution computed tomography (HRCT) is regarded as the gold standard for structural lung imaging, but does not routinely provide much functional information. While contemporary CT-techniques such as Xenon-CT are able to assess regional lung ventilation (6), its use is currently limited to research settings. Ventilation-perfusion (VQ) scintigraphy and single-photon emission computed tomography (SPECT) (7) are other currently available methods of assessing lung ventilation but suffer from low spatial resolution and long acquisition times. Most importantly, unlike magnetic resonance imaging (MRI), all the aforementioned techniques involve ionizing-radiation, limiting its use in patient groups such as pregnant women and children, and in situations where frequent repeated imaging is required even with low-dose CT protocols (8, 9). Despite being radiation-free, lung MRI has historically been challenging for the following reasons: (i) low proton density of lung tissue; (ii) rapid signal decay due to multiple interfaces between air and soft-tissue structures; and (iii) motion artefacts generated by cardiac, vascular and respiratory motion (10). These factors greatly reduce the signal-to-noise ratio of images acquired, resulting in the lungs appearing as dark, signal voids.

Recent advances in lung MRI have expanded its use in certain pulmonary disorders (11). Inhaled contrast agents such as hyperpolarized (HP) gases have permitted the assessment of lung ventilation, microstructure, and alveolar-capillary diffusion (12). Functional lung MRI is also possible using inhaled fluorinated gases (13), oxygen-enhanced techniques (14), and free-breathing proton methods (15, 16). Together, these novel and emerging techniques have generated a wealth of new information regarding the structure–function relationships of various lung diseases.

This review sets out to describe the most common approaches to ventilation imaging using MRI-based techniques. Each section contains a brief overview of the principles and physics behind each imaging modality, followed by a review of its current and potential clinical applications to various lung disease including but not limited to asthma, COPD, interstitial lung disease (ILD), cystic fibrosis (CF) and COVID-19. This review is divided into two main sections. First, we discuss HP-MRI, the most mature and well-established of these methods. Second, we describe the alternate and emerging techniques including fluorinated gas MRI, oxygen-enhanced MRI and free-breathing proton MRI. We conclude by examining the advantages and limitations of various techniques, and consider future directions. As this review is written with the clinician in mind, detailed technical discussions are beyond the scope of this article. Likewise, perfusion imaging will not be covered.

## Hyperpolarised gas MRI

### Basics of hyperpolarization

Hyperpolarization of noble gases involves the transfer of angular momentum from circularly polarized light to the noble gas nuclei, significantly increasing the atomic nuclei alignment. This results in a 10^4^–10^5^-fold increase in the magnetic resonance (MR) signal, enabling gases such as helium-3 (^3^He) and xenon-129 (^129^Xe) to be imaged despite their low levels of intrinsic polarization. Hyperpolarization can be achieved by either spin-exchange optical pumping (17) or metastability exchange optical pumping (18), with the former more commonly used in practice, and polarization levels of ~20–50% easily attainable for ^3^He (19, 20) and ^129^Xe (21, 22). Although this will not be discussed further, the interested reader is referred to references (23, 24) for additional information.

It is worth noting that hyperpolarization is not limited to noble gases and has also been achieved with carbon-13 (^13^C). HP ^13^C MRI allows *in-vivo* probing of enzyme-mediated metabolic processes such as cancers and metabolic diseases, and an excellent review of this topic can be found here (25–27).

### Hardware

Polarizers crucial for the production of HP gas can be custom built (28–32) or purchased from commercial companies such as Polarean and Xemed LLC (33, 34). Dedicated transceiver coils tuned to the resonance frequency of the gas nucleus of interest are also required. MRI scanners must also be upgraded with broad-band capabilities.

### Transportation

Depolarization of HP gas is accelerated by the presence of paramagnetic oxygen, magnetic field inhomogeneities, and atomic interactions between HP gas and the storage cell. The use of specialized transport equipment can overcome these challenges, facilitating long distance transportation of HP gas (35, 36).

### Gas delivery

A typical inhalation mixture consists of 200–300 mL of HP gas diluted with medical-grade nitrogen in a Tedlar bag to make up a 1 L volume. Inhalation occurs from end-expiration via a mouthpiece, with images acquired under breath hold conditions of roughly 10–20 s. Addition of an exhalation circuit facilitates collection and recycling of exhaled ^3^He.

### Safety profile

Both ^3^He and ^129^Xe are extremely safe in the small quantities as used during HP-MRI. Other than possible transient minor oxygen desaturation observed shortly after inhalation, no serious adverse events have been described (37).

Xenon has anesthetic properties at a sustained minimum alveolar concentration of 63–71% (38, 39), but these levels are not attainable with current HP-MRI protocols. Nonetheless, ^129^Xe has been shown to be extremely safe even after inhalation of three times the usual dose, with only mild and fleeting symptoms such as dizziness, paresthesia, euphoria and hypoesthesia being reported (40).

### Noble Gas availability

^3^He has a low natural abundance and is derived primarily from the radioactive decay of tritium (41), but much of its supply is redirected toward usage as a neutron detector (42, 43). ^129^Xe, an isotope of xenon, has a natural abundance of 26%. An enriched ^129^Xe mixture is often used in HP ^129^Xe MRI to help improve the MR signal. Enriched xenon costs about A$310/L compared to A$45/L for 26% ^129^Xe natural abundance mixture (12). The scarcity and exorbitant cost of ^3^He, together with the fact that dissolved phase imaging is exclusively limited to ^129^Xe has prompted the shift toward its use in recent years.

## Pulmonary functional imaging approaches

### Static ventilation imaging

**Figure 1:**
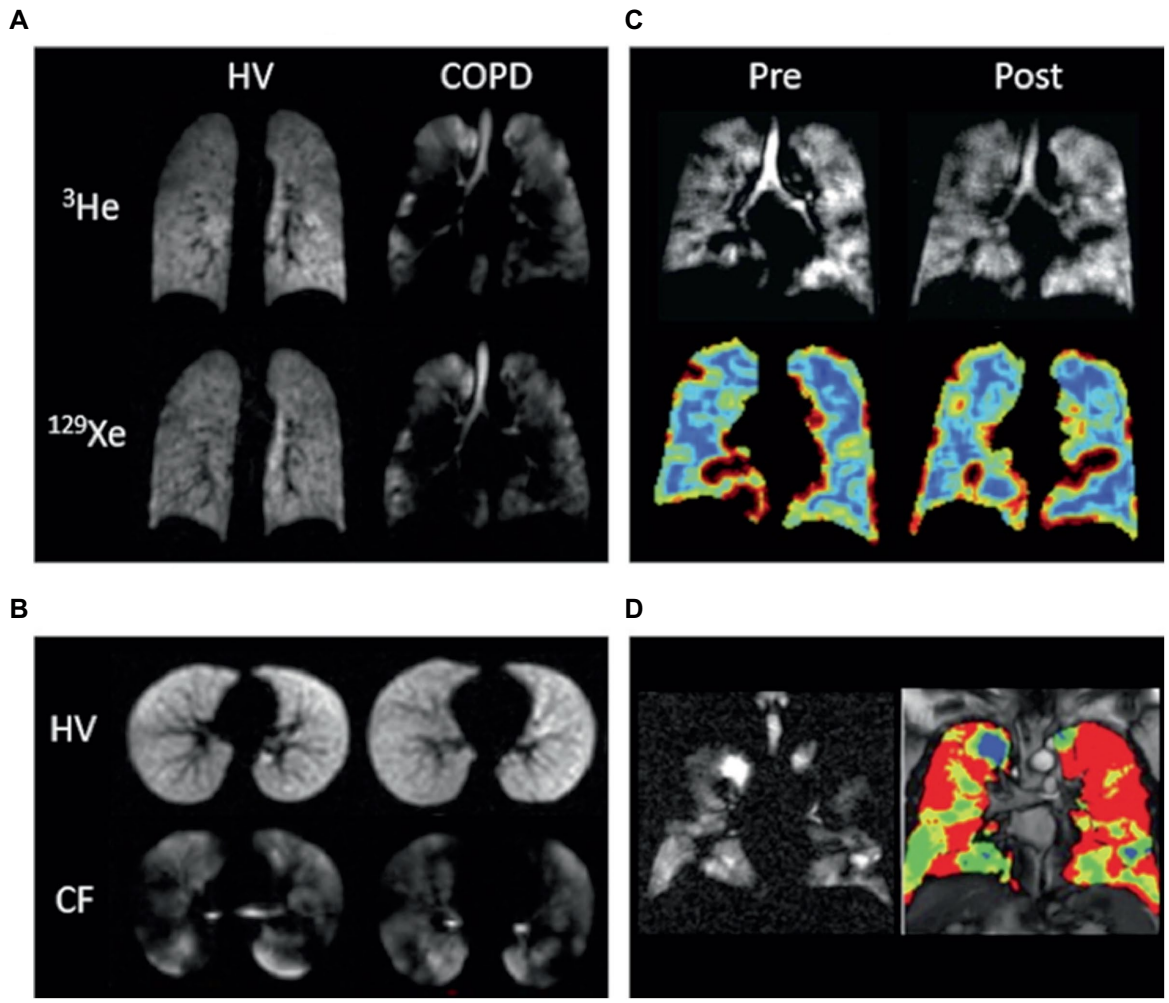
Ventilation imaging. **(A)** ^3^He and ^129^Xe ventilation images of a healthy non-smoker (HV) and a patient with chronic obstructive pulmonary disease (COPD). **(B)** ^129^Xe ventilation images of a healthy 6-year-old (HV, FEV1 = 95%) and an 11-year-old with cystic fibrosis (CF, FEV1 = 102%). **(C)** ^129^Xe ventilation images (top) and coefficient of variation maps (bottom; blue = low COV, red = high COV) of a patient with asthma pre-and post-bronchodilator inhalation. **(D)** ^129^Xe ventilation image (left) and binning map (right; red = defect, yellow = low intensity, green = medium intensity, blue = high intensity) from an older patient with asthma (FEV1 = 53%). In this case, ventilation defect percentage is defined as the ratio of the number of red pixels to the total number of pixels in the whole lung × 100. Adapted with permission from (23).

HP-MRI permits the direct visualization of the distribution and heterogeneity of lung ventilation (Figure 1) (44). Ventilation defects represent areas of signal void (absence of HP gas) and are commonly quantified using ventilation defect percentage (VDP) or ventilated volume percentage (VV%) (45–49). VDP is calculated by dividing the ventilation defect volume (VDV) by the thoracic cavity volume, and VV% represents the inverse of VDP. Ventilation heterogeneity is best assessed using signal intensity binning (48, 50, 51), and ventilation coefficient of variation calculations (52, 53). Due to its increased sensitivity, HP ^129^Xe MRI may identify clinically relevant ventilation defects that would otherwise be missed by ^3^He (54, 55).

### Dynamic ventilation imaging

Compared to static ventilation which provides a snap-shot of the pulmonary gas distribution pattern during a single breath-hold, dynamic ventilation allows us to study the time-dependent distribution of gas within the lungs over the entire respiratory cycle (56–58). As image acquisition begins right before inspiration, a slight modification in the gas delivery process is required, with various protocols in use (56, 57, 59). One such protocol required subjects to inhale the HP gas mixture over the first half, and exhale over the second half of a 15 s acquisition period (56).

Dynamic ventilation is able to provide information on the rate and filling patterns of the central and peripheral airways (60), individual lung lobes, and in some cases, the extent of diaphragmatic excursion. Dynamic ventilation in healthy volunteers is characterized by the uniform distribution of HP-gas throughout the lungs during inspiration, followed by a homogeneous decline in signal intensity on expiration. In contrast, heterogeneous filling pattern of lung lobes, different gas inflow rates and achieved maximum signal intensities, as well as limitation in diaphragmatic excursion due to hyperinflation are some of the findings in those with lung disease (56, 57, 59, 61–63).

Multiple-breath HP gas MRI is in essence dynamic ventilation imaging performed over multiple breath cycles. During each breath, a fraction of the HP gas is replaced by newly arrived gas, and as sequential scans are acquired at each subsequent breath-hold, a volume fractional ventilation measure is calculated by computing the rate of change in HP gas MRI signal intensity during wash-in and/or wash-out breath maneuvers (64–67). As a quantitative measure of regional ventilation, fractional ventilation mapping is able to elucidate delayed ventilation and gas trapping in certain lung diseases.

### Diffusion-weighted imaging

**Figure 2:**
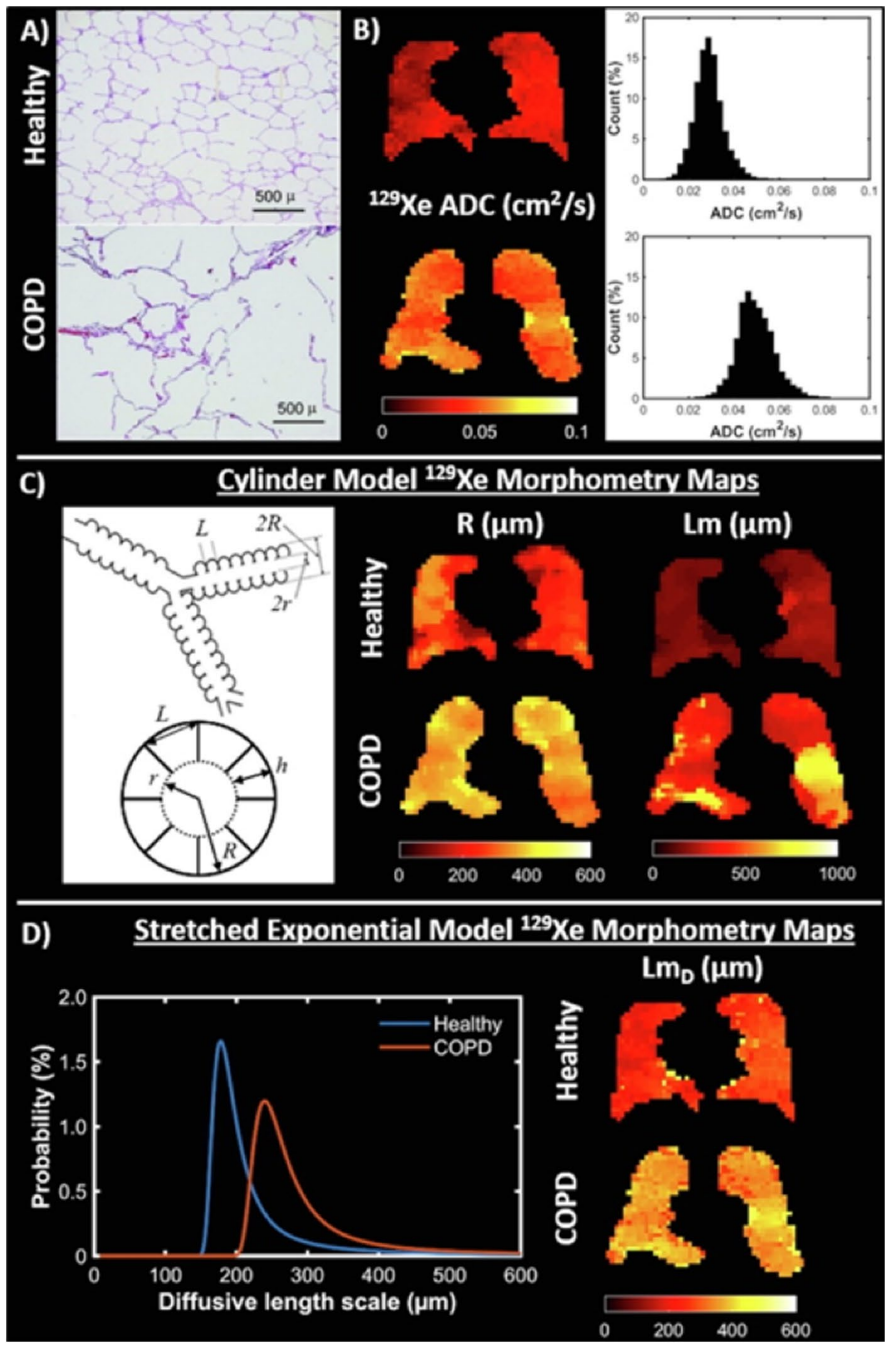
Diffusion imaging. **(A)** Examples of histological slides from a healthy lung (top) and lung with emphysema (bottom) that are used to calculate mean linear intercept (Lm) measurements. **(B)** ^129^Xe ADC maps and whole lung ADC histograms for a healthy volunteer (23-year-old female, top) and chronic obstructive pulmonary disease (COPD) patient (68-year-old male, bottom). **(C)** (Left) Schematic drawing of the cylindrical model of acinar airway geometry based upon the Haefeli-Bleuer and Weibel acinar geometry (73). **(C)** (Right) Cylinder model ^129^Xe lung morphometry maps of acinar airway radius (R) and mean linear intercept (Lm) in the same healthy volunteer and COPD patient as in **(B)**. **(D)** (Left) Probability distributions of diffusive length scale derived from the stretched exponential model for the same healthy volunteer and COPD patient. **(D)** (Right) Stretched exponential model ^129^Xe lung morphometry maps of mean diffusive length scale (Lm_D_) for the same healthy volunteer and COPD patient. Adapted with permission from (23).

Diffusion-weighted imaging (DWI) exploits the high free diffusivity of ^3^He and ^129^Xe (68, 69) to probe the lung microstructure (70). Diffusion is quantified using the apparent diffusion coefficient (ADC), and visually represented on an ADC map with higher values suggestive of enlarged alveoli/emphysema (71, 72). Other acinar airway morphological parameters such as surface-area-to-volume-ratio, alveolar radii and mean diffusive length scale (LmD) can also be derived, and serve as additional biomarkers of lung microstructure (Figure 2) (70, 74–76). Although ADC values obtained using ^129^Xe and ^3^He are not directly comparable, studies have shown them to provide similar information (54, 77, 78).

### Dissolved phase imaging

**Figure 3:**
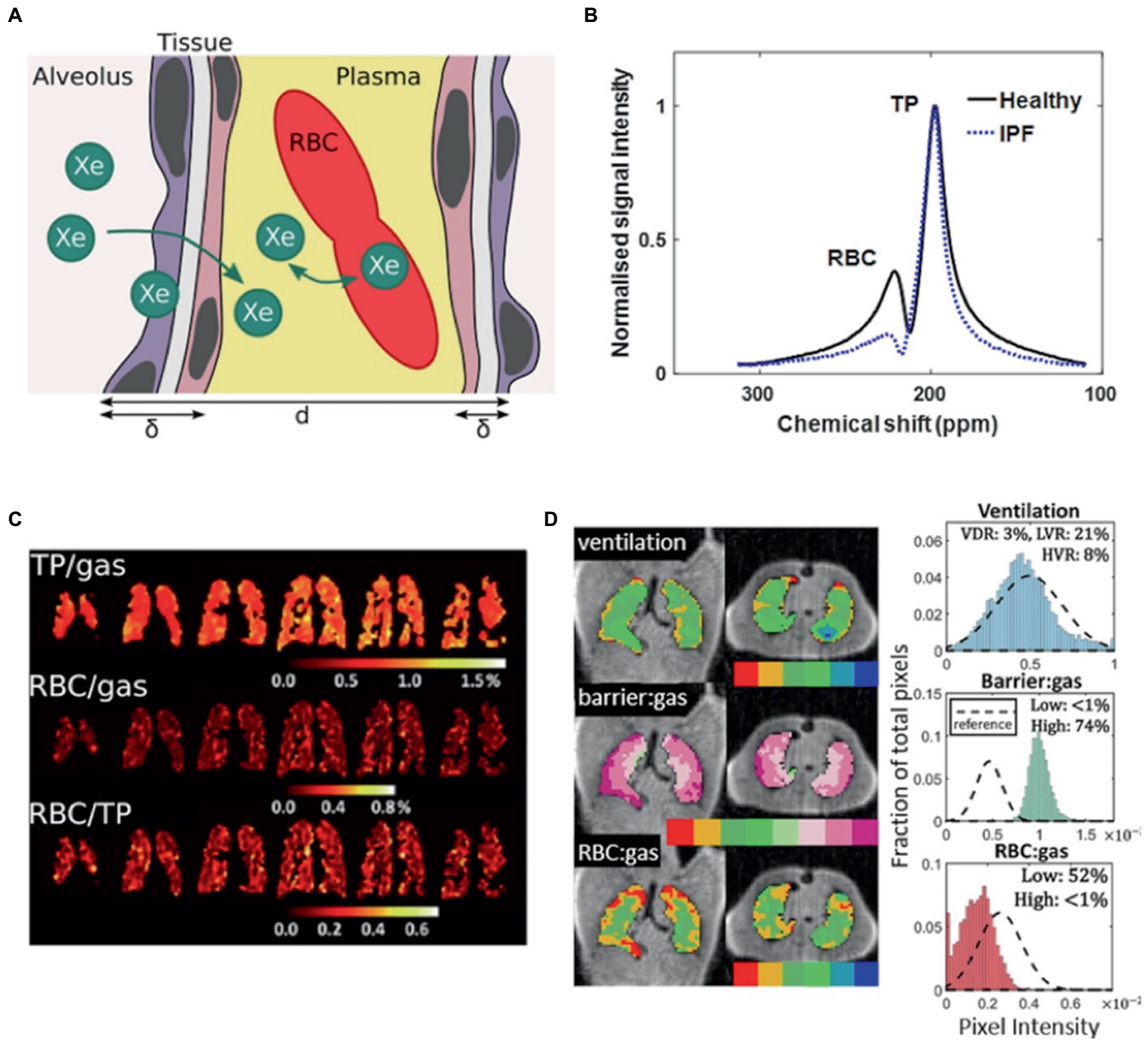
Probing gas exchange. **(A)** Cartoon of diffusive exchange of xenon gas from alveolus to capillary, via the parenchymal tissue barrier. The tissue wall thickness (air-blood barrier thickness) is represented by δ, and the total septal wall thickness separating neighboring alveoli is represented by *d*. **(B)** ^129^Xe MR spectra obtained from a healthy subject (black line) and a patient with idiopathic pulmonary fibrosis (IPF) (blue line). **(C)** Iterative Decomposition of water/fat using Echo Asymmetry and Least-squares estimation (IDEAL) chemical shift imaging of dissolved ^129^Xe in the lungs of a patient with moderate chronic obstructive pulmonary disease, illustrated in the form of ratio maps. **(D)** Representative binning maps and histograms derived from Dixon-based dissolved-phase ^129^Xe MRI acquired from a patient with IPF, highlighting the characteristic high TP (barrier) signal and low RBC signal compared with healthy normal subjects (dashed histogram). (The notation barrier: gas is equivalent to TP/Gas). Adapted with permission from (23).

After inhalation, majority of HP ^129^Xe remains in the gas phase, with ~2% dissolved in the lung parenchyma/plasma (barrier) and blood (RBC). At each of these transitions, ^129^Xe exhibits a chemical shift in its resonance frequency relative to the gas phase by 197 ppm and 218 ppm in the barrier and RBC phase (79, 80). MR spectroscopy (81, 82), chemical shift saturation recovery and chemical shift imaging techniques (22, 83, 84) are able to distinguish these changes, allowing gas-exchange to be quantified as the signal ratios of ^129^Xe within each compartment, or by alveolar morphological parameters such as septal wall thickness (Figure 3) (21, 85–89).

A summary of these biomarkers is provided in Table 1. Reference values are based on currently available data as large-scale population studies are lacking.

**Table 1:** Summary of key biomarkers derived from HP-MRI.

Biomarker	Description of what it measures	Suggested reference values
VDP (VV%)	Ventilation	0–5% (95–100%) (90, 91)
CV	Regional ventilation heterogeneity	Mean CV <15% (92) IQR CV <10% (90)
ADC	Alveolar size	^3^He: 0.1–0.3 cm^2^/s (46, 71, 78, 93–96) ^129^Xe: 0.03–0.04 cm^2^/s (77, 78, 97, 98)
LmD	Alveolar size	^3^He: 212 ± 24 um (99) ^129^Xe: 205 ± 23um (99)
RBC:Barrier	Gas-exchange function and parenchymal tissue thickening	Dependent on imaging technique
RBC:Gas	Gas-exchange and perfusion	Dependent on imaging technique
Barrier:Gas	Tissue thickening	Dependent on imaging technique

VDP, ventilation defect percentage; VV%, ventilated volume percentage; CV, coefficient of variation; IQR, interquartile range; ADC, apparent diffusion coefficient; LmD, mean diffusive length scale; RBC, red blood cell.

## Clinical applications

### Asthma

In the absence of treatment changes or external factors, many of the ventilation defects seen in asthmatics tend to either persist in the same location over time, or intermittently affect predictable spatial locations (19, 49, 100–103) even after an interval of 6 years (104). Furthermore, while the number and size of ventilation defects, and ventilation heterogeneity increases following bronchoprovocation (53, 101, 105–107), the locations of these defects are found to be highly reproducible regardless of the method of bronchoprovocation, suggesting a predilection for certain airways to be affected in asthma (108). Taken together, these findings suggest that ventilation abnormalities in asthma are neither widespread nor homogeneous, but regionally heterogeneous.

Ventilation defects have been associated with airway remodeling and mucus plugging. Using computed tomography (CT), bronchial wall thickening, a hallmark of airway remodeling, and gas trapping, an indirect measure of airflow obstruction, can be directly assessed, and found to correlate spatially and quantitatively with ventilation defects on HP-MRI (109, 110). Likewise, a correlation between higher mucus plugs quantified using CT (111), increased markers of gas trapping, and greater VDP have similarly been reported in asthmatics (112–114), raising the possibility that ventilation defects may be a consequence of proximal airway mucus occlusion with distal gas trapping.

Airway inflammation may also contribute to ventilation defects. Asthmatic subjects with high sputum eosinophils were observed to have a greater number of ventilation defects than those with lower counts (115). Significant correlation between increased ventilation defects and higher blood eosinophil count (116), sputum eosinophilia (115–117), fraction of exhaled nitric oxide (49, 106, 109), and neutrophils on bronchoalveolar lavage (110) have also been reported. How airway inflammation causes ventilation defects remain unclear, and has been postulated to involve increased mucus production and reduced mucus clearance (111, 118, 119). Yet, not all mucus plugs are associated with airway inflammation (114). Similarly, the relative contributions of airway remodeling and mucus plugging toward ventilation defects are difficult to assess, and likely variable between individuals (120).

Clinically, ventilation defects have also been associated with asthma severity (45, 121), poorer asthma control and lower quality of life measures (122), even in those with milder disease (117). Similarly, higher VDP was associated with increased asthma exacerbations requiring hospitalizations (116), and higher exacerbation frequency (117). HP-MRI has also been shown to be more sensitive to asthma disease activity than subjective symptoms and spirometry, with ventilation defects observed in asthmatics with normal lung function who are asymptomatic or minimally symptomatic (19, 45). The minimum clinically important difference for VDV and VDP has recently been proposed (123), but lacks validation. Improvements in ventilation defects and overall ventilation heterogeneity have consistently been described in asthmatic subjects following treatment with bronchodilator therapy (19, 55, 115, 124), montelukast (125), after deep breathing exercises post-methacholine challenge (53), and monoclonal antibodies (126–128).

The relationship between ventilation defects and spirometric indices is complex. While numerous studies have reported significant correlations between the number of ventilation defects and FEV_1_ (45, 49, 55, 105, 109, 116, 117, 121), the ratio of FEV1 to forced vital capacity (FVC) (45, 49, 55, 106, 116, 117, 121, 125), and forced expiratory flow at 25 and 50% interval (45, 49, 125), correlations with FVC (45, 49, 117, 125) and the ratio of residual volume to total lung capacity (49, 106, 121) have not always been found. Ventilation defects have likewise been shown to correlate well with advanced lung function tests such as lung clearance index (122), and forced oscillation technique-measured resistance and impedance (129). Early data also suggests airway closure to be the dominant mechanism for these poorly ventilation regions (51).

It is worth mentioning that regional ventilation abnormalities in asthma have been identified using nuclear scintigraphy from as early as the 1960s (130–133), and later on using SPECT with Technegas (134). Compared to HP-MRI, these methods are limited by their inherently low spatial resolution, long scanning time, and need for ionizing radiation; factors that impede their clinical uptake and translation.

### Chronic obstructive pulmonary disease

COPD patients often demonstrate ventilation defects or regions of ventilation heterogeneity on HP-MRI (46, 47, 93, 94, 135–138). These defects improve with bronchodilator therapy (139), show little intra-day variability but change after 1 week despite stable spirometry (46). Regions of high VDP have also been found to correlate with emphysematous areas on CT (140). When used in conjunction with CT, HP-MRI is able to phenotype severity of COPD (141), and differentiate healthy volunteers from those with disease (142).

Using time-resolved breath-hold ^3^He MRI, Marshall et al. was able to visualize ventilation defects with delayed filling in a small cohort of COPD patients. Based on the pattern of delayed filling, the authors postulated that these represent regions of collateral ventilation (143). A recent study utilizing fast dynamic ^129^Xe MRI sequence has also reported the presence of delayed ventilation in a group of COPD patients, a finding not observed in any of the healthy volunteers in the control group (144). If validated in larger cohorts, dynamic MRI may offer additional insights into the pathophysiology of COPD, assist in the detection and localization of pulmonary air leaks (145), and provide a non-invasive alternative to the assessment of collateral ventilation in patients undergoing bronchoscopic lung volume surgery.

ADC values in emphysematous regions have been found to be ~2 times higher than in healthy lungs (54, 77, 78), and correlate well with emphysema burden on CT (146). Detection of age-related emphysema has also been described (147–151). Importantly, ADC values compare favorably to current gold standard histological measurements of alveolar size (152–154).

Early detection of emphysema has been shown using ADC (155, 156), lung morphometry (157), and alveolar wall thickness (158, 159). As a biomarker of emphysema progression, ADC was observed to increase in a small group of ex-smokers with COPD over a 2-year period despite stable FEV1 (136), making it a potential treatable trait. Intra-and inter-day reproducibility of ADC measurements have also been reported (20, 46, 94).

Compared to spirometry, ventilation biomarkers showed increased sensitivity to changes in regional ventilation (139, 160), bronchodilator therapy (139), and longitudinal lung function decline (136, 161). VDP was also predictive of COPD exacerbation requiring hospitalization (162), and longitudinal changes in St George’s Respiratory Questionnaire (163). Similarly, numerous studies have revealed strong correlations between ADC and FEV1 (78, 95, 157), diffusion capacity of the lung for carbon monoxide (DLCO) (54, 77, 78) and quantitative CT (54, 146, 157, 164). Furthermore, compared to CT derived mean lung density and emphysema index, ADC demonstrated higher sensitivity at separating those with COPD from healthy subjects, and better correlation with DLCO (93, 142).

### Cystic fibrosis

CF is an inherited disorder due to a mutation in the cystic fibrosis transmembrane regulator gene. The lungs are often the primary site of this disease, which is currently incurable. Using HP-MRI, ventilation defects are commonly seen in CF patients and often appear in higher numbers than healthy volunteers (165, 166). These defects are often heterogeneous and patchy, with one study reporting ~5 times more ventilation defects in CF patients (mean FEV1 66% ± 27%) compared to healthy volunteers. Importantly, CF patients with normal FEV1 were also found to have 2–4 times more ventilation defects than healthy volunteers, highlighting the superior sensitivity of HP-MRI over FEV1 (165, 167, 168).

Studies examining the relationship between VDP and spirometry have yielded mixed results, with some studies reporting a high level of correlation (169–171), and others, none (168, 172). This is not surprising given the different physiologic process that VDP and FEV1 measure. In contrast, VDP has been found to correlate well with lung clearance index (90, 166), a marker of ventilation heterogeneity that is more sensitive than conventional spirometry in the detection of mild CF (173); and exhibit the greatest sensitivity in identifying ventilation defects in patients with early CF lung disease when compared to proton lung MRI, lung clearance index (LCI), low-dose CT and spirometry (92). As a marker of disease progression, VDP was best able to identify longitudinal changes in CF patients with normal lung function when compared to spirometry, plethysmography and LCI (174, 175).

HP-MRI has also been used to monitor treatment responses in CF patients. For example, in a two part study evaluating the effect of short-and long-term ivacaftor treatment on ventilation defects in CF patients, a 13 and 9% reduction in VDP was observed after 28 days and 48 weeks respectively, along with improvements in FEV1. Notably, VDP was also observed to improve in patients whose FEV1 remained unchanged, again demonstrating the higher sensitivity of HP-MRI over traditional spirometry (171). As expected, both VDP and FEV1 promptly returned to baseline after cessation of treatment. Similar improvements in VDP have also been reported following antibiotic therapy in CF patients hospitalized with a pulmonary exacerbation (172), and after bronchodilator with albuterol (167). Although studies examining the therapeutic response of chest physiotherapy have failed to demonstrate any overall change in the number of ventilation defects, significant differences in the spatial locations of these defects were noted post intervention (168, 169). Comparable findings have also been reported in CF patients after maximal exercise (176).

Reassuringly, intra-scan and inter-scan reproducibility of HP-MRI images have been demonstrated in stable CF cohorts over intervals ranging from 1 to 64 weeks, with ventilation defects often remaining in the same spatial location (174, 177, 178). To date, there are no studies looking at dissolved phase imaging in *CF*.

### Interstitial lung disease

Most studies examining ILD have focused on dissolved phase imaging. Of the few studies examining ventilation abnormalities in ILD, increased ventilation defects (179, 180) and ventilation heterogeneity (181) have been observed.

HP-MRI DWI is sensitive to the enlarged airways (bronchiectasis) and cystic spaces (honeycombing) present in fibrotic lungs (96). Elevated ADC and LmD have been reported in individuals with idiopathic pulmonary fibrosis (IPF) and found to correlate with DLCO and CT fibrosis score (182). LmD was also noted to increase over a 12-month period while other metrics remained stable, highlighting its potential role in monitoring disease progression (182). Diffusion biomarkers may additionally have a role in differentiating fibrotic from inflammatory ILD (183).

Gas-exchange assessed using whole-lung spectroscopic measurements have revealed significantly lower ^129^Xe RBC to barrier ratio (RBC: Barrier) (82, 184), and increased alveolar septal wall thickness in subjects with ILD compared to healthy volunteers (185), suggesting the presence of diffusion limitation. As spectroscopic methods lack spatial information, dissolved phased imaging was developed (179, 186–188), and repeatedly showed elevated ^129^Xe barrier uptake in those with IPF (179, 188). ^129^Xe RBC transfer was also reduced, and corresponded spatially to areas of fibrosis on CT (22, 188), though correlated poorly with CT fibrosis scores (179). The increased barrier uptake and decreased RBC transfer account for the low RBC:Barrier characteristic of subjects with IPF (22, 179, 186, 188–190).

Diffusion biomarkers correlate strongly with DLCO (82, 179, 184–186, 188, 191), and appear to be more sensitive toward longitudinal disease progression in IPF than current clinical tools (191, 192). Its repeatability has also been demonstrated over time (193, 194).

Emerging evidence support the use of Xe gas-exchange imaging in identifying areas of early/active disease in IPF that are histologically abnormal, but undetected on HRCT (179, 195). If confirmed in future studies, these at-risk regions may be the target of increased monitoring or therapeutic drug trials.

### COVID-19 and other lung diseases

COVID-19 is a novel infectious disease caused by the SARS-CoV-2 virus. First detected in late 2019, it was declared a global pandemic by the World Health Organization in March 2020. Beyond the acute respiratory phase, there is emerging evidence that symptoms can persist for months after the initial infection has resolved. These individuals are said to suffer from long-COVID, with fatigue and breathlessness the two most common complaints (196, 197). Interestingly, investigations such as blood test, lung function tests, or chest imaging often do not reveal any specific explanation for these symptoms (198, 199). It is here that HP-MRI, in particular ^129^Xe MRI, has made an impact on our understanding about the causes and diagnosis of long-COVID.

In a small study of COVID-19 patients, ^129^Xe MRI revealed alveolar capillary diffusion limitation in all subjects 3 months after COVID-pneumonia hospitalization despite normal or near normal CT and DLCO (200). These findings build on an earlier study that examined COVID-19 patients <1 month after discharge (201) and alludes to the possible etiology of persistent respiratory symptoms after the initial infection. Similar findings have also been reported in long-COVID patients who did not require hospitalization (202). In this study by Girst et al., previously hospitalized and never hospitalized patients with long-COVID were both found to have significantly lower RBC-to-barrier ratio compared to healthy volunteers, with no difference found between groups. Given that both groups had normal spirometry, DLCO (though this was lower in the never hospitalized subgroup), and normal/near normal chest CT, these findings suggest that mild COVID-19 disease can result in persistent symptoms and gas exchange abnormalities that are undetected by conventional investigations. In a separate study, similar gas-exchange abnormalities were reported in previously hospitalized long-COVID patients, with additional evidence of small vessel pruning derived from complementary quantitative lung CT analysis (203). Overall, these results are consistent with other findings that implicate alveolar membrane thickening and pulmonary vascular dysfunction (from microthrombi or alteration in pulmonary blood flow) as possible pathophysiologic explanations for long-COVID (204, 205).

Although most attention has been directed toward gas-exchange abnormalities, ventilation defects have also been observed in long-COVID patients, implicating airways disease in the pathophysiology of long-COVID. In one study involving 76 long-COVID and 9 healthy volunteers, VDP was reported to be significantly worse in those with COVID-19 compared to healthy volunteers, and also in patients who were hospitalized at the time of their COVID-19 infection compared to those who were not (206). Furthermore, VDP was also related to 6-min walk distance and exertional SpO2, but not to quality of life or dyspnea scores (206).

^129^Xe MRI has also revealed new insights into our understanding of various other lung diseases such as non-specific interstitial pneumonia (180), inflammatory ILD (183), pulmonary vascular disease (207, 208), and e-cigarette smoking (209).

## Other novel and emerging alternatives

### Fluorinated gas MRI

Fluorine-19 lung MRI (^19^F-MRI) was first performed in humans in 2008 (13), and uses non-toxic and naturally abundant fluorinated gases as contrast agents (210, 211). Unlike HP-MRI, hyperpolarization of ^19^F is not required prior to imaging and dedicated ^19^F hardware, while preferred, is not essential. Extensive signal averaging to improve image quality is also possible (212).

^19^F gases are typically inhaled as a normoxic mixture, with perfluoropropane (PFP; C_3_F_8_) (210) and sulfur hexafluoride (SF_6_) (211) the most commonly used gases. Unlike HP-MRI, image acquisition typically occurs after steady state equilibrium of ^19^F has been reached. This is often achieved by having the subject continuously breath from a large volume Tedlar bag, e.g., 5 L of a mixture of 79% PFP and 21% O_2_ until the bag is empty. Following this, the subject inhales from a separate Tedlar bag containing 1 L of the same PFP-O_2_ mixture (213), with images acquired during a 10–15 s breath-hold. Although image acquisition is also possible without the wash-in period, i.e., subject only inhales a single breath of 1 L PFP-O_2_ mixture, the resultant images are often of lower quality (214).

A homogeneous ventilation pattern is classically seen in healthy subjects (210, 214), with ventilation defects and increased ventilation heterogeneity observed in those with CF (215, 216), asthma (210), COPD (210, 217) and post lung transplantation (210). Furthermore, preliminary data suggests a strong correlation between ^19^F-MRI VDP and FEV1 in a cohort of COPD subjects (217). Intra-and inter-session reproducibility has also been demonstrated (218, 219).

^19^F-MRI is well suited to study wash-in and wash-out kinetics as its MR signal recovers quickly and free-breathing can be performed with normoxic gas mixtures. These properties enable the quantification of regional (215, 220, 221) and collateral ventilation in subjects with lung disease (222). ^19^F has also recently been used to assess lung ventilation and perfusion (223, 224), with VQ-mismatches identified in subjects with COPD (225, 226).

Studies comparing ^19^F-MRI with HP-MRI are limited. A preliminary study involving 5 healthy volunteers showed that VV, VDV and VDP measurements were similar using either ^19^F-MRI or HP-^3^He-MRI despite ^19^F images being of much poorer resolution (227). In contrast, a separate study of 3 healthy volunteers comparing ^19^F-MRI with HP-^129^Xe-MRI reported a lower VV% and CV in ^19^F measurements when compared to ^129^Xe, with the differences attributed to the lower observed signal with ^19^F (228). More recently, McCallister et al. showed that while both ^19^F-MRI and HP-^129^Xe-MRI were able to identify ventilation abnormalities in a small cohort of mild CF patients, these abnormalities were not entirely congruent, suggesting the added utility of ^19^F-MRI in identifying “slowly ventilated regions,” and how VDP obtained from each technique may not be equivalent (229).

### Oxygen-enhanced MRI

**Figure 4:**
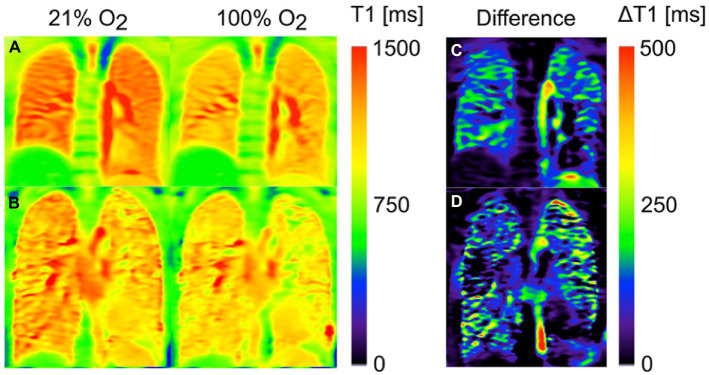
Oxygen-enhanced MRI T1 maps of a healthy volunteer **(A)** and a patient with cystic fibrosis (CF) **(B)** after inhalation of 21 and 100% oxygen. The third column shows the corresponding difference maps: While a homogenous reduction of T1(100% O2) in comparison with T1(21% O2) is found for the healthy volunteer **(C)**, some regions of the CF patient **(D)** show no or only small changes. Assuming that this effect can be mainly ascribed to reduced regional oxygen supply, the difference map can be interpreted as a surrogate for ventilation. Adapted with permission from (234).

Oxygen alters the T_1_-weighted signal intensity of the pulmonary blood circulation (14, 230, 231), permitting indirect imaging of the lungs. During tidal breathing, a series of T_1_-weighted images are acquired using a conventional MRI scanner during both normoxic and pure oxygen conditions (232, 233), with output data represented as T_1_ maps or quantified as relative enhancement ratio or oxygen transfer function (OTF) (Figure 4) (235). Using these metrics, individuals with CF and COPD were found to exhibit a heterogeneous reduction in T_1_ relaxation time compared to healthy individuals (233, 236, 237). In subjects with asthma, oxygen-enhanced MRI (OE-MRI) was sensitive to airway inflammation (238), disease severity, and showed good 1-month reproducibility and intra-observer agreement (239). Differences in T_1_ signal intensity changes were also noted in subjects with a variety of pulmonary diseases including ILD (240).

OE-MRI is able to detect the therapeutic effect of bronchodilators and inhaled corticosteroids in individuals with COPD (241), and been found to be comparable to quantitative CT in assessing pulmonary function loss and disease severity in individuals with COPD (242, 243), asthma (244) and connective tissue disease-ILD (245). Moreover, in subjects undergoing lung volume reduction surgery, OE-MRI was shown to be at least equivalent, if not superior to multidetector CT and SPECT in the evaluation of post-operative clinical outcomes (246). OTF has also been proposed as a potential early marker of chronic lung allograft dysfunction (247). Despite underestimating VDP, OE-MRI was reported to correlate well with ^3^He VDP (248). VQ assessment using OE-MRI has also been described (14, 249).

### Free-breathing proton MRI

The development of proton MRI functional lung imaging was driven by the need for a more accessible method of assessing lung function that did not involve complex experimental set-up, or additional costly equipment such as dedicated transmit-receiver coils and multi-nuclear capable MRI scanners.

Early researchers showed that it is possible to image the lung using MRI (without any additional special equipment or contrast agents) if a fast acquisition sequence is combined with a low magnetic field and non-rigid image registration. By applying simple signal subtraction, regional lung ventilation could be quantified, and ventilation maps generated (250).

Fourier-decomposition MRI (FD-MRI) is an innovative approach that permits the simultaneous imaging of lung ventilation and perfusion (15). FD-MRI works on the principle that changes in lung density during the respiratory and cardiac cycle create an oscillation in the MR signal that is converted into ventilation and perfusion-weighted maps by Fourier transformation (250, 251). As with all proton MRI lung imaging techniques, successful FD-MRI relies on the use of low TE sequences (below 1 msec) to reduce signal decay, and non-rigid image registration to compensate for the changes in the shape of the lungs throughout the respiratory cycle (15).

FD-MRI is able to identify ventilation defects in subjects with asthma and COPD, with FD-MRI VDP correlating strongly with ^3^He MRI VDP (252, 253). FD-MRI VDP was also found to decrease post salbutamol and increase after inhaled methacholine in asthmatics (253), and correlated well with pulmonary function test and CT measurements in those with COPD (252). A strong correlation between FD-derived fractional ventilation and ^19^F washout has also been described (254), and reproducibility previously established (255).

Phase-resolved functional lung MRI (PREFUL) is another unique approach that shares similarities to FD-MRI (16), with both methods providing an indirect quantification of ventilation and perfusion based on oscillations of the MR signal intensity within the lungs of a freely breathing subject. Whereas FD-MRI uses only the signal change amplitude to quantify these measures, PREFUL also considers the phase-component and hence the name “phase-resolved” functional lung imaging. The result is a set of phase-resolved ventilation and perfusion cycles with an increased temporal resolution compared to conventional FD-MRI.

Several ventilation and perfusion parameters can be derived from PREFUL ventilation and perfusion maps. These include regional ventilation, ventilation derived by cross-correlation, VDP, perfusion (arbitrary units), quantified perfusion, perfusion defect percentages and VQ match maps (16, 256–258).

**Figure 5:**
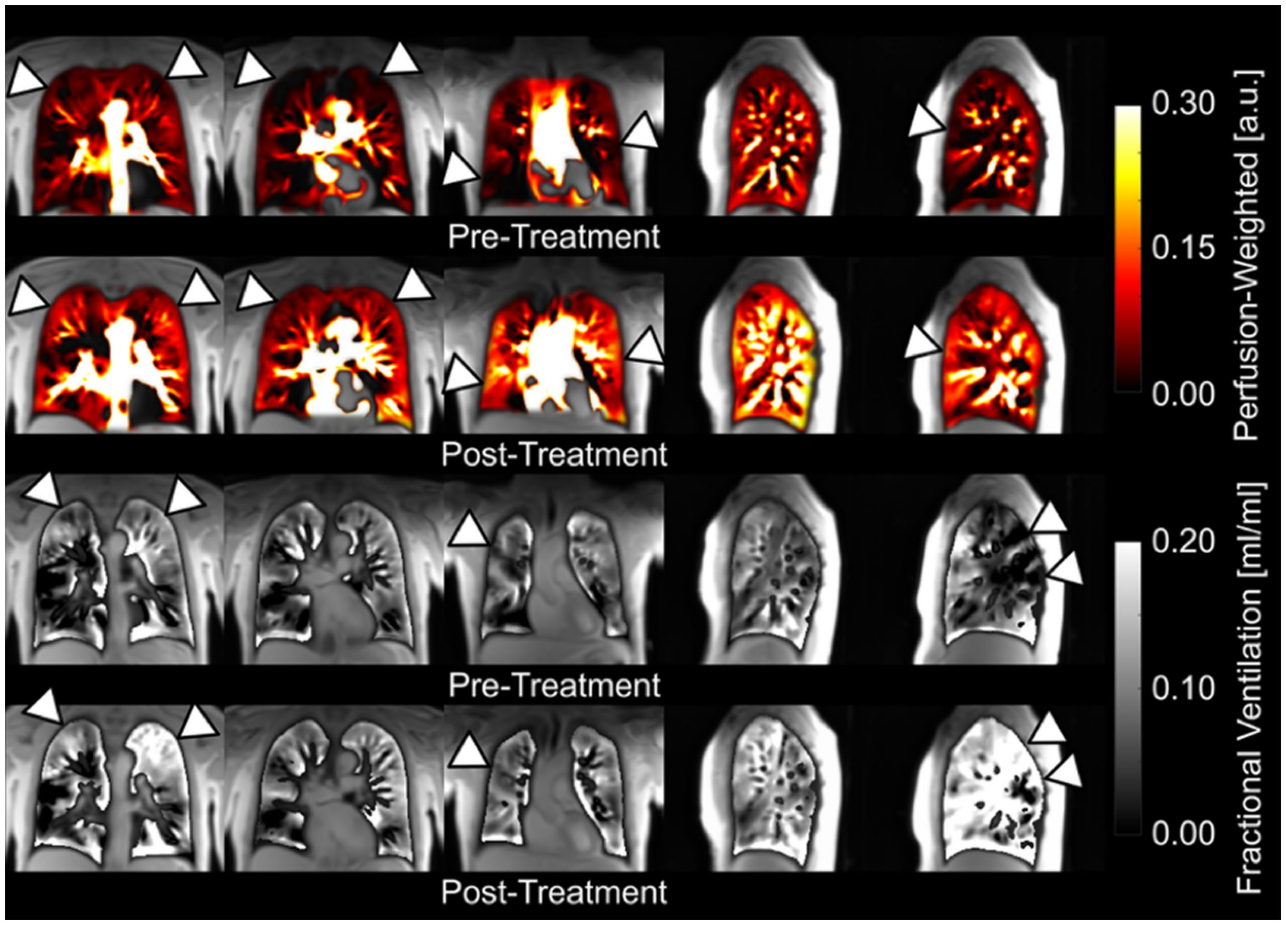
Coronal and sagittal fractional ventilation and perfusion-weighted maps of a patient with chronic obstructive pulmonary disease obtained with a free-breathing, contrast agent-free method (PREFUL) pre-and post-14 days of inhaler treatment (264). Please note the visible improvements (arrowheads) in perfusion and ventilation after treatment and the possibility for a pixelwise ventilation-perfusion assessment. Adapted with permission from (234).

In the feasibility trial, PREFUL was able to distinguish healthy individuals from those with lung disease (16). COPD patients were subsequently showed to have an increased number of ventilation and perfusion defects, greater ventilation heterogeneity, and higher VQ mismatch compared to healthy volunteers (258, 259). PREFUL was also able to discriminate healthy CF patients from those experiencing an exacerbation (260), and track changes in regional ventilation following treatment (259, 261). PREFUL derived regional flow volume loops also showed increased sensitivity to early stages of chronic lung allograft dysfunction (CLAD) in subjects post lung transplantation (257), and was predictive of CLAD related death or transplant loss in a large prospective cohort (262). In contrast, conventional flow volume parameters failed to show any significant difference between healthy lung transplants and early-stage CLAD (263). Likewise, PREFUL has been successfully used to study post-treatment changes in those with COPD (Figure 5) (259) and chronic thromboembolic pulmonary hypertension (CTEPH) (265), and show good agreement when compared to SPECT, dynamic contrast-enhanced-MRI, and ^129^Xe MRI (260, 266–268).

The described techniques have so far been limited to 2D imaging although 3D modalities such as 3D PREFUL (269) and SENCEFUL (270) have recently been developed, and show promise. For instance, early 3D PREFUL data suggests a strong correlation between 3D ventilation parameters and spirometric measurements (269, 271). Additionally, repeatability of this method has also been demonstrated (258, 271). As these methods are in the early phases of development, further research is needed before they can be translated to clinical practice.

Without detracting too much from the focus of this review, PREFUL derived perfusion measures have been used to study a variety of cohorts including healthy subjects and those with COPD, CTEPH and CF (16, 256, 258, 272, 273). Validation against dynamic contrast-enhanced MRI (256, 273, 274), and repeatability have also been demonstrated (258).

## Conclusion and outlook

In this review, we discuss four unique methods of functional lung imaging using MRI: (1) HP-MRI; (2) ^19^F-MRI; (3) OE-MRI; (4) FD-MRI and PREFUL. HP-MRI is a well-established, safe and tolerable method for assessing lung function that is considered the functional lung MRI reference standard. By directly visualizing and quantifying the inhaled gas distribution, HP-MRI provides a direct assessment of lung ventilation in contrast to OE-MRI, FD-MRI and PREFUL. HP-MRI has exhibited immense potential in (i) phenotyping disease, (ii) assessing treatment response, (iii) early detection, and (iv) longitudinal monitoring. The high sensitivity (55, 275–277) and repeatability of HP-MRI biomarkers may enable future clinical trials to be undertaken with smaller sample sizes (125, 171). Emerging evidence also support its role in guiding bronchial thermoplasty (278, 279) and placement of endobronchial valves (280, 281). While diffusion-weighted and gas-exchange imaging are at an earlier stage of development, we believe they will develop into its full potential over time. Standardization of scanning protocols is essential before multi-center trials can be conducted, with guidance provided by the ^129^Xe MRI Clinical Trials Consortium (282). While ^129^Xe MRI has been approved for routine clinical use in the United Kingdom since 2015 (283), it remained limited to clinical research in the United States up until December 2022 when it received United States Food and Drug Administration approval for use in ventilation imaging (33). These represent important milestones in its broader adoption. The fact that a typical ^129^Xe MRI examination can be easily undertaken within 5–15 min allows for seamless integration into current imaging workflows, although the high setup cost (due to expensive polarizers, dedicated coils and xenon gas), complex imaging protocol, and need for skilled personnel (to run the polarizer, administer the xenon gas and acquire images) has limited its use to dedicated research institutes and may pose barriers to future uptake. While the development of a portable ^129^Xe hyperpolarizer shows promise as a cost-effective platform for wider clinical dissemination of ^129^Xe MRI (284), additional unanswered questions remain about how such a service will be funded, where they will be located, which patient groups should be referred, what constitutes normal values, and in what format results will be conveyed to referring clinicians. Looking ahead, ^129^Xe MRI may be used to distinguish a range of cardiopulmonary disorders (186, 189, 190) by analyzing cardiogenic signal oscillations arising from ^129^Xe dissolved in the pulmonary circulation (81). The complementary use of other imaging modalities may likewise create powerful diagnostic tools to enhance our understanding of various lung diseases such as COVID-19 (201, 203, 285).

^19^F-MRI has been touted as a possible alternative to HP-MRI given it also provides a direct measure of ventilation but at a lower operational cost – expensive polarizers are not required and fluorinated gases are less costly than HP-gases. Furthermore, due to its rapid signal recovery and ease of administration as normoxic gas mixtures, ^19^F-MRI excels in multi-breath imaging and the study of wash-in and wash-out kinetics. Despite these advantages, the wider adoption of ^19^F-MRI has been hindered by several factors. Firstly, ^19^F-MRI is currently restricted to ventilation imaging (286–290), with ^19^F lung diffusion weighted MRI still in the pre-clinical phase (288–290). Secondly, ongoing technical advances focusing on optimizing image acquisition, image quality and improving signal-to-noise ratio is required. Thirdly, rigorous reproducibility and validation studies in various patient groups are lacking, and would be needed to build up the evidence base for its use. Lastly, in this era of climate change, one must acknowledge that inert fluorinated gases, which are also utilized in many other industries, are potent greenhouse gases with long lifetimes (291). While the contribution from ^19^F-MRI is likely to be small, efforts to capture and recycle exhaled ^19^F gases, similar to those available for HP gases, should be pursued (292). As ^19^F-MRI technology advances, there is strong potential for its wider clinical use.

OE-MRI also suffers from poor image and spatial resolution. More importantly, interpretation of results can be challenging due to the influence of supplemental oxygen on pulmonary physiology, and the difficulty in teasing out which of ventilation, perfusion, or diffusion is responsible for the signal changes (237, 293). Long acquisition times are also problematic, and substantial work is still required to define its role in pulmonary functional imaging.

FD-MRI and PREFUL offer a promising, cost attractive, and patient friendly alternative to functional lung imaging as imaging can be undertaken on a standard MRI scanner without the need for any additional costly equipment, or breath-holding maneuvers that are often challenging for patients with lung disease (272). The short scanning time of a couple of minutes is also beneficial for less cooperative patients such as children (294). These attributes have enabled PREFUL to be successfully employed in the assessment of healthy, and premature infants with bronchopulmonary dysplasia, without the need for procedural sedation (295, 296). Compared to HP-MRI and ^19^F-MRI, FD-MRI and PREFUL provide an indirect assessment of ventilation. Although ventilation biomarkers derived from these methods appear to show less sensitivity to diseased states, there is overall good agreement with HP-MRI (253, 260, 261, 268). Free-breathing MRI also has the advantage of producing detailed perfusion maps (273, 297, 298), a feature that is lacking with the other modalities. While image acquisition with free-breathing MRI is relatively easy, considerable post-processing analyses is required to generate regional functional maps. Despite this, among all the other described techniques, free-breathing MRI has possibly the lowest barrier to entry. This will be a major advantage in its scalability and transition to the clinic. A summary of the key differences between these techniques can be found in Table 2.

**Table 2:** Summary of the key differences between techniques.

	HP-MRI	^19^F-MRI	OE-MRI	FD-MRI/PREFUL
Implementation				
Set-up cost	++++	+++	+	+
Skilled personnel	++++	+++	+	+
Post-processing	++++	++++	++++	++++
Aspects of lung function assessed				
Ventilation	Yes	Yes	Yes*	Yes*
Microstructure	Yes	No	No	No
Gas exchange	Yes	No	Possibly^†^	No
Perfusion	No	No	Possibly^†^	Yes
Patient factors				
Breath-hold requirement	Yes^‡^	Yes^‡^	No	No
Scanning time	+	++	+++	+
Ionizing radiation	No	No	No	No
Image resolution				
Spatial resolution	++++	+++	+	+++
Temporal resolution	NA	NA	+	++++

^19^F, fluorine-19; FD, Fourier decomposition; HP, hyperpolarized gas; MRI, magnetic resonance imaging; OE, oxygen-enhanced; PREFUL, phased-resolved functional lung.

* These methods can only provide an indirect measure of ventilation.

† Signal derived from OE-MRI represents a combination of ventilation, diffusion and perfusion.

‡ Limited or no breath hold may be possible with dynamic ventilation.

Compared to other functional imaging techniques such as CT, VQ and SPECT, the biggest advantage of the abovementioned MRI-techniques is that they are all free from ionizing radiation. This make these methods friendly for use in vulnerable populations such as children and pregnant women, as well as in longitudinal studies where repeated imaging is required. Moreover, there is now a growing body of evidence that these functional MRI techniques are more sensitive than current clinical endpoints such as spirometry, and may one day be used in its place.

In conclusion, novel MRI approaches to functional lung imaging offer a range of powerful and creative tools to interrogate lung function in ways that surpass current clinical methods. Despite being at different stages of maturity, these techniques all show tremendous potential in helping us better understand the structure–function relationships in a variety of lung diseases.

## Author contributions

DL, BT, and FT: conceptualization. CF: data curation and writing of original draft. CF and FT: formal analysis. CF, DL, BT, and FT: review and approval of final version. All authors contributed to the article and approved the submitted version.

## Funding

CF is the recipient of a Monash University post graduate scholarship.

## Conflict of interest

The authors declare that the research was conducted in the absence of any commercial or financial relationships that could be construed as a potential conflict of interest.

## Publisher’s note

All claims expressed in this article are solely those of the authors and do not necessarily represent those of their affiliated organizations, or those of the publisher, the editors and the reviewers. Any product that may be evaluated in this article, or claim that may be made by its manufacturer, is not guaranteed or endorsed by the publisher.
